# Inflammatory bowel disease and periodontitis: A retrospective chart analysis

**DOI:** 10.1002/cre2.609

**Published:** 2022-06-15

**Authors:** Nazia Abrol, Sharon M. Compton, Daniel Graf, Pallavi Parashar, Giseon Heo, Monica P. Gibson

**Affiliations:** ^1^ School of Dentistry University of Alberta Faculty of Medicine and Dentistry Edmonton Alberta Canada

**Keywords:** Crohn's disease, inflammatory bowel disease, periodontitis, ulcerative colitis

## Abstract

**Objectives:**

This study examined the variation in prevalence of periodontitis among different sexes, age groups, smoking status, and oral hygiene adherence in patients affected by either Crohn's disease (CD) or ulcerative colitis (UC).

**Materials & Methods:**

This study was a retrospective chart analysis that collected data from the School of Dentistry's Oral Health Clinic at the University of Alberta, Edmonton, Canada. Patients' electronic health records between the years of 2013 and 2019 were analyzed. Multiple keywords such as IBD, CD, UC, and periodontal disease with various spelling combinations were used for searching and gathering pertinent data, which was then further assessed. After applying the inclusion and exclusion criteria, a total of 80 patient charts were included. These patient charts were thoroughly screened to gather information such as age, sex, smoking status, and a variety of periodontal parameters. Collected data were analyzed using SPSS software by using Pearson's χ^2^, Pearson's correlation, and Mann–Whitney *U*‐test.

**Results:**

IBD had an impact on the severity of periodontitis in patients between the ages of 50 and 64 years with higher odds ratio (OR). Biological sex or history of smoking in IBD patients did not have higher odds of developing periodontitis. Plaque score derived from this retrospective study was used to estimate the patient's oral hygiene status and showed no impact. Also, prevalence of periodontitis did not differ between UC and CD. We anticipated some of these findings because of the retrospective nature of the study.

**Conclusions:**

Within the limitation of the retrospective study, IBD patients in the 50–64 age group years showed a higher odds ratio for a greater prevalence of periodontitis. Thus, a closer periodontal recall and evaluation in these patients is recommended for early diagnosis and preventive care. It is advised that periodontists work closely with gastroenterologists to maintain periodontal health in IBD‐affected individuals.

## 1 INTRODUCTION

Periodontitis is mostly a chronic inflammation and leads to/involves the degeneration of tissues surrounding and supporting the teeth (Ridgeway, 2000). In most cases, this inflammatory process begins as gingivitis that can progress to periodontitis in a susceptible host (Ridgeway, 2000). Exposure to dental plaque biofilms and biofilms that accumulate on the tooth surfaces is considered critical and the primary event (Ridgeway, 2000). However, an array of host‐related factors can impact disease susceptibility, severity, and rate of progression (Genco, 1992; Heitz‐Mayfield, 2005). These risk factors often directly impact host immunity, thereby making individuals more prone to periodontitis. Because of this, there is considerable variation among individuals in their risk for disease progression, prevalence, and severity (Beck, 1994).

Periodontitis shares genetic and environmental etiological factors with other diseases and disorders and, therefore, affected individuals can show predisposition to several or all of those diseases (Albandar et al., 2018). Over the last years, susceptibility to periodontal disease has been studied in the context of chronic obstructive pulmonary disease, chronic kidney disease, cardiovascular disease, and cognitive impairment (Brandtzaeg, 2001; Gil‐Montoya et al., 2017; Katancik et al., 2005; Loos et al., 2000; López‐Jornet et al., 2012; Renvert et al., 2010; Scannapieco & Panesar, 2008; Tonetti & Van Dyke, 2013). The relationship between the gastrointestinal tract and oral cavity has also gained attention. IBD is a broad term used to define a group of disorders affecting the gastrointestinal tract (Vavricka et al., 2013). CD and UC are two main forms of IBD (Vavricka et al., 2013). They present with chronic intestinal and systemic inflammation, resulting from an aberrant mucosal immune response to the bacteria of the gastrointestinal tract in susceptible individuals (Vavricka et al., 2013). Canada has one of the highest incidence rates of IBD in the world (Kaplan et al., 2019). This prevalence of IBD in Canada thus affects the quality of life of a significant proportion of the total population (Kaplan et al., 2019; The impact of inflammatory bowel disease in Canada, 2012).

It has been proposed that periodontitis and IBD share common etiological factors (Van Dyke et al., 1986). Pathogenesis model of periodontitis and IBD have been proposed that provide a plausible connection between microbial composition, environmental factors, and/or genetic factors (Brandtzaeg, 2001; Figueredo et al., 2011; Van Dyke et al., 1986). Although the etiology of IBD is quite unclear, however several hypothesis have been laid out by several researchers. Some are of the opinion that IBD is mediated by chronic inflammation triggered via environmental stimulus in a genetically susceptible individual. Microbiological connection (genus *Wolinella*, *T. denticola*) has also been reviewed as a potential factor that might be accountable for the altered predisposition to periodontitis in IBD patients (Brito et al., 2013; Van Dyke et al., 1986). A higher prevalence of *Treponema denticola* and other bacteria, in connection with opportunistic infections in subgingival sites, was reported in IBD patients (Brito et al., 2013). Immune‐inflammatory response studies demonstrated elevated levels of TNF‐α (tumor necrosis factor‐alpha) in the gastrointestinal tract of CD patients, as well as in the gingival crevicular fluid of periodontitis patients (Figueredo et al., 2011). A cohort study was conducted to explore the association between IBD and periodontitis. The study concluded higher prevalence, severity, and extent of periodontitis in IBD patients when compared with non‐IBD individuals (Vavricka et al., 2013). Several efforts are continuously being made to explore the association of these two conditions.

Given the high prevalence of IBD in Canada in the absence of a clear genetic etiology, we asked if this connection is also seen in a population with higher susceptibility to IBD. We present a retrospective study aimed to estimate the risk of developing periodontitis considering variables like sex, age, smoking status, oral hygiene adherence in a population affected by different forms of IBD, that is, CD or UC.

## 2 MATERIALS AND METHODS

### 2.1 Study design and data collection

This study evaluated patient records from the School of Dentistry's Oral Health Clinic consisting of a history of IBD (IBD, Inflammatory bowel syndrome, CD, UC, indeterminate colitis) between January 2013 and December 2019. All data for this study were obtained from existing axiUm clinic records. AxiUm Dental is a HIPAA‐compliant (Health Insurance Portability and Accountability Act), ONC‐ATCB (Office of the National Coordinator‐Authorized Testing and Certification Body) certified system. It involves applications such as electronic health records (EHR), billing, and practice management designed to address the needs of educational programs in dentistry. No personal data, including name, that could reveal the patient identity was accessed. Since the study was designed to access the complete pool of patients who visited the School of Dentistry clinic from January 2013 to December 2019, unique keywords were used to define the search in the patient records (Table 1). The screening process is displayed in Table 2. The inclusion criteria consisted of patient records with a diagnosis of IBD, CD or UC, ages ≥20 years, and complete periodontal assessment records. The exclusion criteria consisted of incomplete periodontal assessment charts, if selected patient records did not indicate IBD, CD, or UC or like terms (Table 2). Exclusion criteria: Patients with a history or ongoing use of recreational drugs such as cannabis, cocaine, hallucinogens, and methamphetamine; patients taking bisphosphonates as it masks the bone loss; pregnant and lactating patients and immunocompromised individuals; patients <20 years of age to rule out aggressive forms of periodontitis.

**Table 1:** Keywords for screening charts

Inflammatory Bowel disease	Ulcerative colitis	Crohn's disease
Inflammatory bowel disease	Ulcrative colitis	Crohns disease
Inflammatory bowel disease	Alcerative colitis	Crohn
Inflammatory bowel disease	Ullcerativ collitus	Chron disease
Inflamatry bowel disease	Ulcrative colittis	Chrons disease
Inflamtry boul disease	Elcertive colitis	Crohon disease
Bowel dsease	Ullcerative colitis	Cron disease
IBD	UC	CD
Inflammation bowel disease	Allcrativ colitis	Crohns disiase
Inflammatory bovel disease	Ulcer colitis	Cronss disease
Inflammatory boul disease	colitis	Craun disease
Bowel disease	collitis	Crauhn disease

**Table 2:** Flow diagram presenting the screening process

Records identified through database searching (*n* = 239)
Records after duplicate removal (*n* = 132)
Records screened (*n* = 132)
Records excluded (*n* = 52)
Records eligible (*n* = 80)
Final charts reviewed (*n* = 80)

Patient charts that fulfilled the selection criteria were comprehensively reviewed. Information on the type of intestinal disease, that is, IBD, CD, or UC was collected. Sex and age were recorded. Variables collected included the number of missing teeth (*n*), maximum periodontal probing depths (PD), percentage of bone loss (expressed in percentage), clinical attachment loss (expressed in millimeters), mobility of tooth (Miller Mobility Grade I, II, III), and furcation involvement (Hamp furcation class I, II, III) (Fukuda et al., 2008; Hamp et al., 1975; Miller, 1950). Bone loss was radiographically determined by subtracting 2 mm from the cementoenamel junction (CEJ) and measuring up to the alveolar bone crest (X). The percentage bone loss was calculated by dividing X with length from CEJ to the apex of the tooth multiplied by 100 (Brito et al., 2013). All calculations were done in axiUm software using radiographs available in the patient records. Periodontal charts were analyzed to determine the presence and severity of periodontitis. If absent (healthy periodontium), it was denoted as N. If present, it was recorded as Y and the stage of periodontitis (Stages I–IV) was noted according to World Workshop 2017 (Tonetti et al., 2018). Oral hygiene adherence was estimated from plaque scores due to the retrospective nature of the study. Plaque scores (using the O'Leary plaque scoring method) were recorded on four tooth surfaces, three on buccal (mesial, mid, distal) and one on palatal/lingual. The scores range from 0 to 1; 0 means no plaque, and 1 represents the presence of plaque. The percentage of plaque is calculated by dividing the total surfaces with score one by the total number of surfaces recorded multiplied by 100 (O'Leary et al., 1972). Lastly, the patients' smoking status was recorded as current smokers, past smokers, or non‐smokers. Parameters recorded from each patient chart are listed in Table 3.

**Table 3:** Patient chart parameters

Type of disease: IBD, CD, UC
Age of the patient
Sex of the patient
Number of missing teeth
Maximum probing depth
Maximum bone loss
Maximum CAL
Mobility with 3 grades, 1, 2, 3
Furcation Involvement with 3 grades 1, 2, 3
Plaque percentage
Smoking status: nonsmoker, past smoker, current smoker

### 2.2 Data analysis

Once all the data were retrieved, grouping was done to conduct statistical analysis. Records were divided according to periodontal severity (Stages I–IV). For comparison of biological sex data were grouped into males (denoted as M) and females (denoted as F). For age groupings, patients were divided into five groups as per the NHANES (National Health and Nutritional Examination Survey) criteria: Group 1: included patients from 20 to 34 years of age, Group 2: 35–49 years, Group 3: 50–64 years, Group 4: 65‐74 years, and Group 5: equal to or greater than 75 years of age (Borrell & Talih, 2012). Patient records were grouped according to smoking status. Furthermore, records were divided according to the type of IBD, that is, UC or CD.

The IBM SPSS 21 (Statistical Package for the Social Sciences) statistical package was used for all the statistical analyses. Odds ratio (OR) and 95% confidence intervals (CI) were used to present the risk of periodontitis. A two‐sided *p* < .05 was considered statistically significant. 2 × 2 contingency tables were drawn, and the odds ratio was calculated to determine the sex prevalence for periodontitis in IBD patients. 3 × 2 contingency tables were drawn, and the odds ratio was determined if smoking had any effect on the development of periodontitis in IBD patients. To determine which age group was most affected, 5;× 2 contingency tables were drawn and the odds ratio was calculated. Pearson's correlation was calculated to ascertain if oral hygiene adherence affected the severity of periodontitis in IBD patients. Mann–Whitney *U* test was conducted to verify the effect of disease type, that is, UC or CD, on the development of periodontitis. All tests of significance were evaluated at a 0.05 error level.

## 3 RESULTS

### 3.1 Sex differences

We considered patient records of 43 females and 37 males. In all, 74.4% of females and 75.7% of males presented with periodontitis (Table 4). Pearson's *χ* ^2^ test was used to determine if there is a sex prevalence for IBD gave a *p *value of 0.897, which indicates no significant prevalence.

**Table 4:** Crosstabulation for sex distribution

	Perio disease		
	N	Y	Total
Sex			
F			
Count	11	32	43
Expected count	10.8	32.3	43.0
% within sex	25.6%	74.4%	100.0%
% within perio disease	55.0%	53.3%	53.8%
% of total	13.8%	40.0%	53.8%
M			
Count	9	28	37
Expected count	9.3	27.8	37.0
% within sex	24.3%	75.7%	100.0%
% within perio disease	45.0%	46.7%	46.3%
% of total	11.3%	35.0%	46.3%
Total			
Count	20	60	80
Expected count	20.0	60.0	80.0
% within sex	25.0%	75.0%	100.0%
% within perio disease	100.0%	100.0%	100.0%
% of total	25.0%	75.0%	100.0%

*Note*: Sex × perio disease crosstabulation.

### 3.2 Age variation

Patient records per age group were not uniformly distributed. Age Groups 1 and 2 had seven patient records each, age Group 3 28 patient records, age Group 4 21 records, and age Group 5 17 records (Table 5). The highest number of patients with periodontitis was in age Group 3 (50–64 years) (Table 6), Pearson's *χ* ^2^ test to determine if there were significant differences in incidence of periodontitis between the five different age groups computed the *p *value of 0.009, which implies a significant difference in copresenting periodontitis and IBD between the age groups.

**Table 5:** Periodontitis in different age groups

	Perio disease		
	N	Y	Total
Age group			
1	4	3	7
2	4	3	7
3	3	25	28
4	3	18	21
5	6	11	17
Total	20	60	80

**Table 6:** Cross‐tabulation presenting periodontitis versus age

	Perio disease		
	N	Y	Total
Age group			
1			
Count	4	3	7
Expected count	1.8	5.3	7.0
% within age group	57.1%	42.9%	100.0%
% within perio disease	20.0%	5.0%	8.8%
% of total	5.0%	3.8%	8.8%
2			
Count	4	3	7
Expected count	1.8	5.3	7.0
% within age group	57.1%	42.9%	100.0%
% within perio disease	20.0%	5.0%	8.8%
% of total	5.0%	3.8%	8.8%
3			
Count	3	25	28
Expected count	7.0	21.0	28.0
% within age group	10.7%	89.3%	100.0%
% within perio disease	15.0%	41.7%	35.0%
% of Total	3.8%	31.3%	35.0%
4			
Count	3	18	21
Expected count	5.3	15.8	21.0
% within age group	14.3%	85.7%	100.0%
% within perio disease	15.0%	30.0%	26.3%
% of total	3.8%	22.5%	26.3%
5			
Count	6	11	17
Expected count	4.3	12.8	17.0
% within age group	35.3%	64.7%	100.0%
% within perio disease	30.0%	18.3%	21.3%
% of total	7.5%	13.8%	21.3%
Total			
Count	20	60	80

Odds ratios among different age groups (Table 7) showed consistent higher odds of periodontitis with ages ≥50 years compared to those less than 50 years of age.

**Table 7:** Odds ratio in different age groups

Group 2 versus Group 1 = 1.00
Group 3 versus Group 1 = 11.10
Group 4 versus Group 1 = 8.00
Group 5 versus Group 1 = 2.44

### 3.3 Smoking status

Pearson's *χ* ^2^ test was applied to determine a significant difference between two groups divided based on their smoking status, past and current smokers as one group and nonsmokers as the second group. A *p* = .698 was determined, which implies no significant difference in these groups in presenting with periodontitis in IBD patients.

### 3.4 Oral hygiene adherence

Pearson's correlation test was conducted to evaluate the variation in severity of the periodontitis based on plaque percentage. The analysis revealed no statistically significant difference (*p = *0.339), suggesting that the presence of plaque did not lead to a prevalence of periodontitis in individuals with IBD.

### 3.5 Disease type

Mann–Whitney *U* test was used to establish if there was a difference in periodontitis presentation between the two forms of IBD (UC and CD). With a *p *value of 0.420 no significant difference was observed.

### 3.6 Summary of results

We found no sex predilection for periodontitis in patients presenting with IBD. When patients were grouped into five groups according to NHANES criteria (Borrell & Talih, 2012) IBD patients in the 50–64‐year age range were most affected by periodontitis, with Stage 3 periodontitis being most prevalent in this age group. Smoking in IBD patients appeared not to increase the odds of developing periodontitis. Oral hygiene adherence did not affect the prevalence of periodontitis in patients presenting with IBD. The prevalence of periodontitis did not differ between patients with either UC or CD.

## 4 DISCUSSION

The present study examined incidence and severity of periodontitis in IBD individuals. We found no difference in incidence between the two sexes. Previous studies indicated that males have a higher risk for periodontitis; thus, a sex predilection has been determined to be associated with periodontitis (Eke et al., 2015; Heitz‐Mayfield, 2005). A sex prevalence was also previously noted between patients with IBD depending on the type of IBD (UC and CD) (Rossomando et al., 1990). A study that looked at sex variation in IBD stated that in patients with CD, a greater prevalence of females with the disease was noticed, while in patients with UC, no significant differences between prevalence of the disease in females or males were observed (Brant & Nguyen, 2008). It was also stated that the sex ratios in CD varied with age and geographic region (Brant & Nguyen, 2008). In spite of the male predilection for periodontitis and female predilection for CD, our study failed to establish any sex predilection for the two diseases. A likely reason for this difference is the comparatively small sample size in this study. A larger study with more patients in each age group of both the sexes is needed to explore this further.

According to the NHANES study periodontitis has a higher incidence in the elderly (age groups over 65 and 75) (Billings et al., 2018; Eke et al., 2015). Our study showed the highest odds of presenting with periodontitis in the age range of 50–64. This group had a higher risk of presenting with periodontitis (OR 11.10) when compared to ages 20–34 years. NHANES data similarly established that the prevalence of periodontitis was positively associated with age (Billings et al., 2018; Eke et al., 2015). Literature supports the association between IBD and periodontitis on account of their similar etiologies, that is, dysbiotic microbiota, deregulation of the immune response, and chronic inflammation in genetically susceptible individuals (Lira‐Junior & Figueredo, 2016; She et al., 2020). The association stated thus corroborates with the increased odds of periodontitis in the elderly age group, since age is a risk factor for periodontitis itself and with a history of IBD, the odds may tend to increase (Flemmig et al., 1991; Indriolo et al., 2011; Lira‐Junior & Figueredo, 2016; Papageorgiou et al., 2017; She et al., 2020; Van Dyke et al., 1986). In the present study no linear relationship between the patient's age and these two disease conditions was observed. This could be due to the fact that periodontitis tends to peak in the elderly group, that is, >50 years (Billings et al., 2018). IBD has a bimodal incidence pattern, with the main peak of occurrence between 15 and 25 years of age and a second, smaller rise in IBD occurrence during the fifth to seventh decades of life (Johnston & Logan, 2008). It is likely that patient records included our study represented this second peak of IBD presentation and thus formed most of the data. explaining the increased odds of periodontitis in this age group. On the other hand, another it might also be the result of the cumulative effect of age‐related periodontal changes and peak occurrence of IBD. A study with equal group distribution of patients for each age group should be able to address this.

Smoking is an established risk factor for periodontitis and tends to increase the prevalence and severity of the disease (Bergström et al., 2000; Haber et al., 1993; Kibayashi et al., 2007; Linden & Mullally, 1994; Tomar & Asma, 2000; Van Winkelhoff et al., 2001). The results of the present study did not have the same finding. This could be because the smoking group comprised both past smokers and current smokers.

It is well established that the number of years of smoking and years of cessation are crucial elements in determining the effects of smoking on developing periodontitis. The negative effects of smoking can be observed for up to 11 years following cessation (Tomar & Asma, 2000). There is also an established dose–response between how many cigarettes per day were being consumed and disease severity (Tomar & Asma, 2000). These correlations were not observed in our study. Due to the nature of the study, years of cessation and years of smoking, as well as the number of cigarettes could not be taken into consideration. Thus, there was expected variability in periodontal disease presentation.

We also attempted to assess oral hygiene adherence with the odds of developing periodontitis. We used the plaque score as proxy as this was a retrospective study. We did not observe an effect of plaque on the rates of periodontitis in IBD‐affected individuals. It should be noted that the plaque score might not be a good indicator. If oral hygiene adherence were to be recorded in person using standardized established questionnaires, it would have been assessed differently. This was not possible in this retrospective chart review (Oral Health Questionnaire, 2020).

There was no significant difference in periodontitis incidence in patients with UC versus CD. Therefore, it can be derived that although the clinical presentation of CD and UC vary in terms of periodontitis, there is no variation between these two forms. This could be explained by the fact that both forms of IBD share the common pathogenesis with periodontitis and thus no difference in prevalence of periodontitis was seen in the present analysis.

### 4.1 Limitations

The present study had a few limitations. The first and foremost being the nature of the study design. Retrospective data mining limited the review of patient charts, since the data available were not originally designed to collect information for research conducted. This also limited the number of patient records included, since incomplete patient chart records with missing baseline data were excluded from the study. Secondly, there was a variation in sample size in subgroups, especially in age groups.

## 5 CONCLUSION

The key finding of this study is that patients in the 50–64 years age group with IBD have significantly greater odds of developing periodontitis. Since age has a significant effect on IBD‐affected individuals in developing periodontitis and its severity, it is advised to keep them under regular recall for early diagnosis and maintenance. No sex predilection for periodontitis in patients presenting with IBD was seen. The present study did not state increased odds of periodontitis in patients with IBD with a smoking history. When oral hygiene adherence was assessed in the present study, it did not affect the occurrence of periodontitis in patients presenting with IBD. However, since plaque is an etiological factor in periodontitis, plaque control regimes should be encouraged. Although there is a variation in the clinical presentation of UC and CD, the prevalence of periodontitis did not differ between UC and CD. Patients who have either UC or CD should be examined with the same assessment measures for determining their regime for periodontal care. Since the increased prevalence of periodontitis was seen in IBD, it is advised for gastroenterologists to collaborate with periodontists and possibly other dental professionals to ensure periodontal health is maintained in IBD‐affected individuals.

## AUTHOR CONTRIBUTIONS

Nazia Abrol: contributed to conception, design, data acquisition, and interpretation, performed all statistical analyses, drafted, and critically revised the manuscript. Monica P. Gibson: contributed to conception, design, data acquisition, and interpretation, drafted, and critically revised the manuscript. Sharon M. Compton: contributed to data acquisition and critically revised the manuscript. Daniel Graf, Pallavi Parashar, and Giseon Heo: contributed to data acquisition and critically revised the manuscript. All authors gave final approval and agree to be accountable for all aspects of the work.

## CONFLICT OF INTEREST

The authors declare no conflict of interest.

## ETHICS STATEMENT

This study was a retrospective study that received ethical approval from the University of Alberta Research Ethics and Management Online (REMO) ID#Pro00090612.

## Data Availability

The author elects to not share data.
